# Early Detection for Dengue Using Local Indicator of Spatial Association (LISA) Analysis

**DOI:** 10.3390/diseases4020016

**Published:** 2016-03-29

**Authors:** Mayra Elizabeth Parra-Amaya, María Eugenia Puerta-Yepes, Diana Paola Lizarralde-Bejarano, Sair Arboleda-Sánchez

**Affiliations:** 1 Grupo de Análisis Funcional y Aplicaciones, Universidad EAFIT, Carrera 49 No. 7 Sur-50, Medellín 050022, Colombia; meliza0520@gmail.com (M.E.P.-A.); mpuerta@eafit.edu.co (M.E.P.-Y.); paola.lizarralde@gmail.com (D.P.L.-B.); 2 Grupo Biología y Control de Enfermedades Infecciosas—BCEI, Universidad de Antioquia, Calle 70 No. 52-21, Medellin 050010, Colombia

**Keywords:** dengue risk classification, early warning, spatial analysis, temporal indices

## Abstract

Dengue is a viral disease caused by a flavivirus that is transmitted by mosquitoes of the genus *Aedes*. There is currently no specific treatment or commercial vaccine for its control and prevention; therefore, mosquito population control is the only alternative for preventing the occurrence of dengue. For this reason, entomological surveillance is recommended by World Health Organization (WHO) to measure dengue risk in endemic areas; however, several works have shown that the current methodology (aedic indices) is not sufficient for predicting dengue. In this work, we modified indices proposed for epidemic periods. The raw value of the epidemiological wave could be useful for detecting risk in epidemic periods; however, risk can only be detected if analyses incorporate the maximum epidemiological wave. Risk classification was performed according to Local Indicators of Spatial Association (LISA) methodology. The modified indices were analyzed using several hypothetical scenarios to evaluate their sensitivity. We found that modified indices could detect spatial and differential risks in epidemic and endemic years, which makes them a useful tool for the early detection of a dengue outbreak. In conclusion, the modified indices could predict risk at the spatio-temporal level in endemic years and could be incorporated in surveillance activities in endemic places.

## 1. Introduction

Dengue is the most significant viral disease transmitted by arthropods around the world. It is distributed in tropical and subtropical zones and affects approximately 50–100 million people annually in over 100 endemic countries, where about half of the global population lives [1]. In the Andean region of Latin America, Colombia is the country most affected by dengue [2] and represents an economic cost of around $79.17 per ambulatory case and US $733.32 per hospitalized case [3]. In this country, all places below 1800 m can support *Aedes aegypti*, which is the main vector of the Dengue virus (DENV) in America [4].

Colombia has been considered free of the disease since the 1970s due to vector eradication, but the epidemiological landscape has changed slowly over time, with a subsequent reinfestation by *Ae. aegypti*. Since the 1980s, dengue outbreaks have begun to be common in some Colombian municipalities, and severe dengue epidemics spread rapidly across the country. Dengue epidemics occur every two or three years and have had a strong socio-economic impact on Colombia [5]. 

Due to the absence of a specific treatment or vaccine for the control and prevention of the disease, the WHO recommends maintaining mosquito populations at low proportions, with the aim of reducing vector-human contact. As risk indicators, endemic places use entomological parameters as aedic indices, which measure the number of recipients positive for immature stages and the number of sampled houses. While these indices are useful for measuring risk in some places [6,7,8,9], in other places, it is not possible to establish a positive relation between dengue case occurrence and aedic index values [10,11,12]. To address this, several authors have proposed indices based on pupal stage detection [13] as the best way of quantifying the population of adult mosquitoes, which are responsible for transmission. However, it is very difficult to quantify the number of pupae because this stage lasts only for a short time and is not likely found in the breeding populations.

These facts have led other authors to exclude entomological information and to develop indices based only on dengue cases, which, indirectly, provide evidence of the presence of infected mosquitoes. In [14] three indices were developed that measured dengue risk in Taiwan and characterized the epidemic that occurred in 2002. Then, [15] used a different classification methodology based on the standard deviation of the indices to develop a threshold, and they argued that the new classification was more accurate at establishing a risk scale than that proposed by [14]. However, even though these approximations describe the epidemic behavior and how to classify the risk of dengue occurrence, any of them can be used for early detection of dengue outbreaks in an endemic period. Taking into account the difficulties of establishing the relation between entomological and epidemiological data in some places, the aim of this study was to evaluate the indices developed by [14], which use only epidemiological information, in epidemic and endemic years. To do so, we selected as our study area a Colombian municipality that was endemic for dengue, for which correlations between entomological and epidemiological data were not observed [11].

## 2. Materials and Methods

### 2.1. Study Site

**Figure 1:**
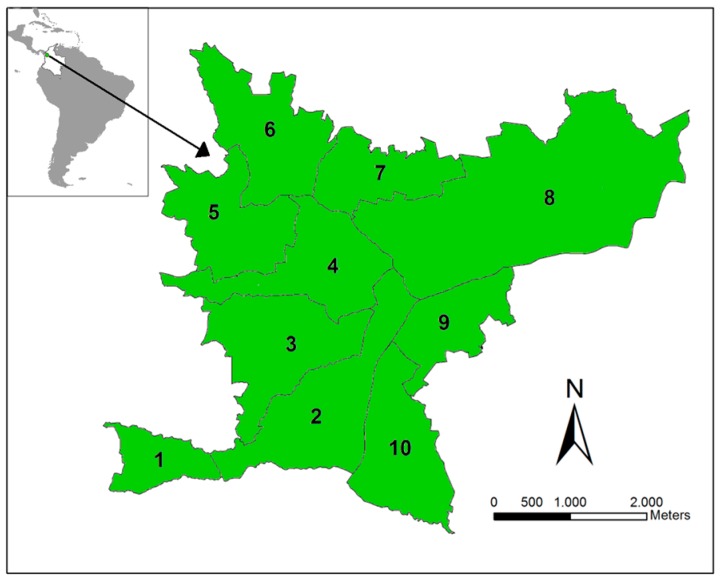
Study site. The Bello municipality is located in Antioquia Department (Colombia). This municipality is divided into 10 main zones in which dengue is endemic.

The study site is the municipality of Bello, which is located in Aburrá Valley (Antioquia, Colombia, Figure 1). We selected this region because it was endemic for dengue and showed co-circulation of the four dengue serotypes over the last 10 years (Henry Pulido, Bello Epidemiologist, personal communication). In 2010, Bello had an estimated urban population of 387,000 over an area of 19.7 km^2^ divided into 82 townships, grouped into 10 zones. Bello is located at an inclined plane, with altitudes ranging from 1600 to 1400 m, with an average annual temperature of 26.7 °C and ~1347 mm of average rainfall. Limited variation is observed in the climatic characteristics among the townships. The municipality has an *Ae. aegypti* presence and suitable climatic factors for its maintenance and, thus, DENV transmission. We consider each of the 10 zones as a unit in our analysis.

### 2.2. Epidemiological Data

Epidemiological data were obtained from Sistema Nacional de Vigilancia en Salud Pública (SIVIGILA) [16]. Since 2010, dengue has been classified into three categories: dengue, severe dengue, and mortality by dengue [16]. We used dengue and severe dengue cases that were classified as probable and confirmed according to laboratory and epidemiological nexus. Case-occurrence data were provided to us at the patient address level. From the total dengue cases reported, most of them could be geo-referenced: 114 of 199 occurred in 2008, 121 of 156 in 2009, 1216 of 1676 in 2010, 130 of 164 in 2011, and 195 of 213 in 2013. Dengue cases were geo-referenced using the patient’s address and Google Earth software with a spatial precision of 20 m or finer [17].

### 2.3. Temporal Indices to Measure Dengue Risk

#### 2.3.1. Literature Indices

To estimate dengue risk in the Bello municipality, we used temporal risk indices developed by [14], which were applied to an epidemic in Taiwan. These indices refer to the frequency, (α), duration, (β), and intensity, (γ), which are useful for evaluating the magnitude and severity of the epidemic event. Briefly, the indices are defined as follows:

The frequency index (α), also called the occurrence probability, measures how often the disease occurred:

(1) $$\alpha =\frac{\mathrm{EW}}{\mathrm{TW}}$$

where EW denotes the number of weeks with a case occurrence and TW is the total number of weeks during the entire epidemic period.

The duration index (β) estimates the average number of weeks in an epidemic wave. An epidemic wave is defined as the number of weeks where cases successively occur:

(2) $$\beta =\frac{\mathrm{EW}}{\mathrm{EV}}$$

where EV is the total number of epidemic waves during the entire epidemic period.

The intensity index (γ), which measures the epidemic severity, is calculated by dividing the incidence rate by the total number of epidemic waves:

(3) $$\gamma =\frac{\mathrm{IR}}{\mathrm{EV}}$$

where IR is the incidence rate during the defined epidemic period. IR is the number of dengue cases that occurred divided by the population of the place.

#### 2.3.2. Modified Indices

We modify the indices α, β, and γ proposed by [14] for dengue epidemics for measuring risk in endemic periods. [14] assumed that TW is the total number of weeks during the entire epidemic period; this period can be more or less than one year, but for this index to be applied to endemic regions, we set this value to one year (represented in epidemiological weeks); therefore, our new α is:

(4) $$\alpha_{1}=\frac{\mathrm{EW}}{\text{one year in epidemiological weeks}}$$

In [14], the EV is the number of sequential weeks with case occurrence, and the dengue risk is inversely related to the number of epidemiological waves. However, in endemic periods, several EV can occur, with possible outbreaks in some of them, which are important for predicting the beginning of the epidemic. We, therefore, change the EV parameter by (maxEV + EW) to ensure that zones with more than one EV can be classified as having a real risk for dengue. With the modification, the new β is:

(5) $$\beta_{1}=\frac{1}{2}\left(\mathrm{MaxEV}+\frac{\mathrm{EW}}{\mathrm{TW}}\right)$$

where MaxEV is defined as the maximum epidemiological wave. In this case, β_1_ refers to the amplitude of a different EW in a particular year:

(6) $$\gamma_{1}=\text{IR}\times \beta_{1}$$

The new γ_1_ refers to the altitude of the epidemiological waves, which denotes the dengue incidence at a specific moment. For comparison purposes, the original indices in the literature and the modified indices were calculated taking into account one year (52 or 53 epidemiological weeks).

### 2.4. Risk Classification

Even though each index indicates a risk of dengue occurrence, it is necessary to establish a threshold that shows if a particular zone has high or low risk. To determine the appropriate methodology for classification, we examine two methods: Local Indicators of Spatial Association (LISA), which was used by [14], and Standard Deviation (SD), which was used by [15].

#### 2.4.1. LISA

The LISA methodology can identify significant spatial clusters [18]. LISA is defined as:

(7) $$I(i)=\frac{\left(X_{i}-\bar{X}\right)}{\delta }\times \sum_{j=1}^{n}[W_{\text{ij}}\times \frac{\left(X_{j}-\bar{X}\right)}{\delta }]$$

where I(i) is the LISA index for region i, $W_{\text{ij}}$ is the proximity of region i to region j, X_i_ is the value of the temporal index of region i, X_j_ is the value of the temporal index of region j, $\bar{X}$ is the average value of the temporal index, δ is the standard deviation of X_i_, and n is the total number of regions to be evaluated.

The term $W_{\text{ij}}$ describes the proximity of region i to region j. If region i is next to region j, a value of 1 is assigned; otherwise, a value of 0 is assigned. For any region i next to region j, the term (X_i_ − $\bar{X}$) × (X_j_ − $\bar{X}$) describes the degree of similarity in a tested index within a designated area and its neighbors.

**Figure 2:**
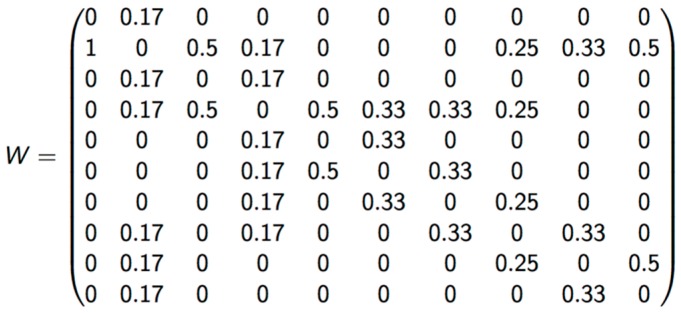
Standardized weight matrix.

The criterion of vicinity used to calculate LISA was Queen, which considers polygons that share any border as neighbors. Matrix $W_{\text{ij}}$ was standardized by dividing it by the number of neighborhoods in each zone (Figure 2).

LISA can identify zones with high (H) or low (L) values according the values of the surrounding areas. It can take values from −1 to 1, representing the correlation grade between one specific zone and the zones in its vicinity. With LISA, it is possible to identify five classes of spatial grouping: HH (zone with high risk surrounded by zones with high risk), LH (zone with low risk surrounded by zones with high risk), LL (zone with low risk surrounded by zones with low risk), HL (zone with high risk surrounded by zones with low risk), and non-significant correlations among risks assigned to each vicinity zone. This significance was calculated using the Monte Carlo methodology [19] for each temporal index for 2010 at 2012, and was identified as high with a 95% statistical significance.

To obtain a final index, we combined the risk for each index and assigned a value of 1 for L and 2 for H, and then calculated their sum. Values corresponding to γ_1_ were assigned a value twice that of the assigned value because this index is composed of α_1_ and β_1_ (Table 1). The capacity of prediction with LISA’s classification was measured by lineal correlation using Spearman's coefficient: risk classification of a particular epidemiological period, defined as four consecutive epidemiological weeks (for example period I in 2008) was transformed to a dummy variable (HH: 4, HL:3, LH:2, LL: 1) and used as the independent variable; as a dependent variable, we used the number of cases occurring in the posterior epidemiological period (for example period II in 2008) in each zone. This analysis was made using R software [20]. 

#### 2.4.2. Chen’s Classification

All zones in the study area were classified as high- (H) or low- (L) risk areas, individually based on their deviation from the means of each index. Given an index, when the value was larger than the mean plus 1.5 × Standard Deviation (x + 1.5 × SD), the zone was classified as high risk (H); otherwise, the zone was classified as low risk (L) [15].

#### 2.4.3. Scenarios to Validate the Modified Indices

**Figure 3:**
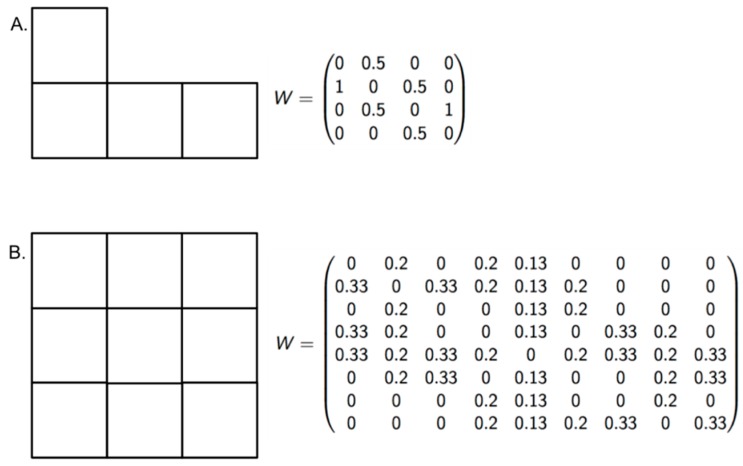
Vicinity configuration and standardized weight matrix. With the aim of validating the modified indices, two block configurations (**A** and **B**) were built to propose scenarios with high dengue incidence.

To measure the sensitivity of the new indices at recognizing different levels of risk, we proposed a null hypothesis that the indices cannot differentiate risk when the dengue incidence is high in all zones of an endemic area, despite variations in EW and EVmax. The number of populations at risk and the number of dengue cases were set to 100,000 and 100, respectively. The parameter EW ranged between 29 and 51, and the EVmax ranged between 10 and 52 (Figure 3, Table 2). If the LISA classification differentiated the risk among the zones despite high incidence values, the null hypothesis was rejected.

## 3. Results

### 3.1. Temporal Indices for Early Detection of Dengue

For this work, the frequency indices (α) calculated using the methodology proposed by [14] and our indices had the same values because we fixed the denominator for both. Comparisons among the values of dengue incidence *vs.* all indices indicated that α was positively correlated with dengue cases. The modified duration and intensity indices (β_1_ and γ_1_) showed high correlation values with dengue, which were not observed with the original indices. Index γ_1_ was not correlated with dengue cases that occurred in 2008, but this is an isolated result with respect to the other years (Table 3).

When β and γ where modified, we found that the observed endemic behavior in each zone was represented by these indices, showing that the modification was useful for identifying areas at real risk of dengue occurrence (Table 3).

### 3.2. Risk Classification

**Figure 4:**
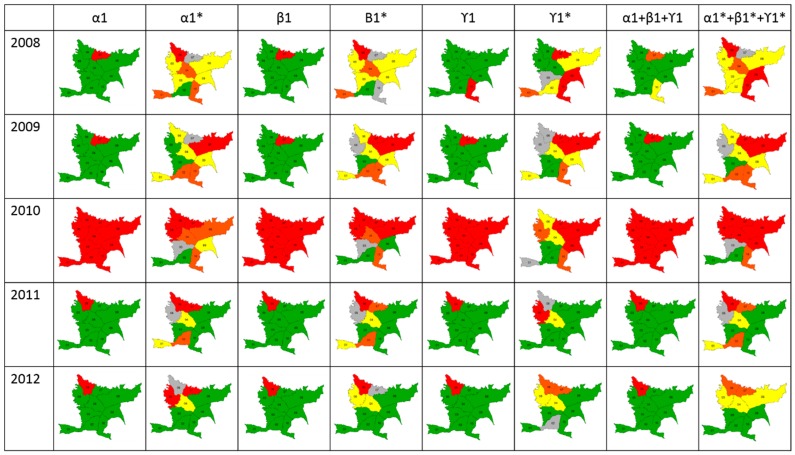
Scale of risk. Maps show the classification of each index and the merged indices using the Standard Deviation (SD) and LISA methodologies (*).

Classification using the SD and LISA methodologies showed that combined indices using LISA could be used to measure risk in endemic areas, whereas the SD methodology was useful only for epidemic years (Figure 4). Differences were observed in thresholds calculated using the LISA methodology because these were lower than Chen’s threshold. Additionally, LISA classifies a specific area based on two aspects: its proper risk and the risk of surrounding zones. For this reason, a spatio-temporal risk of dengue as an early warning could be predicted only using LISA.

Results of lineal correlation between LISA classification and dengue cases occurrence showed that, in average, Spearman coefficient was 0.72 in 2008, 0.70 in 2009, 0.76 in 2010, 0.73 in 2011, and 0.85 in 2012 (*p* < 0.05).

### 3.3. Index Validation

The results of the index validation in endemic areas with high incidence showed that using the LISA classification could possibly lead to identifying a risk scale (Table 2), which indicates the performance of these indices in other epidemiological landscapes and highlights the importance of the temporal scale of occurrence as well as the continuity of occurrence in measuring dengue risk in a specific area. Therefore, we reject the null hypothesis that in cities of high incidence the modified indices represent high risk because they can classify zones according to a risk scale.

## 4. Discussion

Dengue is considered an important vector-borne disease and a serious public health problem in tropical and subtropical countries [21]. From 2008 to 2012, 1657 dengue cases were reported in Bello municipality (Colombia), which, based on the regular behavior of the disease in this location, exceeded the response capacity to control the disease. Bello has 10 main divisions (zones) in which a variable number of neighborhoods are grouped. Indices proposed by [14] were applied to these 10 zones over five years (2008–2012) to identify dengue risk zones across space and time. We find that these indices are not accurate in detecting risk in endemic or epidemic years in Bello (Table 3). Although [15] implemented indices developed by [14] by modifying only the system of classification, it was not enough to detect differential risk across space. Therefore, we concluded that the classification system was not the only reason for the issues related to risk detection; we mathematically modified these indices to improve their capability to predict dengue in different scenarios (endemic and epidemic).

Of these results, we deduced that some of the parameters evaluated were not appropriate for measuring risk in endemic periods and, therefore, we considered that [14] states that dengue risk is inversely related to the number of epidemiological waves; however, this is not applicable to endemic periods because a large wave longitude (temporal duration) is not necessarily responsible for outbreaks. It is clear that short epidemiological waves can also produce dengue outbreaks. In this sense, we took into account the largest wave amplitude in a year plus the number of weeks involved in these waves. The results obtained showed that the modified indices had a greater capacity for classifying dengue risk both in endemic and epidemic years, and our results correspond to the epidemiological data from Bello (Table 3). Therefore, we modified the β and γ indices because they are calculated using the epidemiological wave value. 

After evaluating the performance of the modified indices, we merged them to build a scale of risk. We first used the methodology implemented by [15], which uses standard deviation to classify the risk; however, this methodology is not sensitive enough to detect variation in risk across space and time (Figure 3). We then calculated the risk scale for the modified indices merged using the LISA methodology, which takes into account the values of the surrounding areas. With these results, we checked that the calculated scale was comparable with the epidemiological landscape for dengue in Bello (Table 3). Additionally, it is necessary to have a very fine classification because the idea behind this type of methodology is to reduce the cost of interventions by focusing on risk without neglecting zones that may be epidemiologically important. With our results, the indices can be classified into four categories that indicate low, moderate, high, and very high risk, which is a didactical form that can be used and understood by health authorities in each endemic place. Modified indices can also be used in other endemic places because they are insensitive to differences in dengue incidence, as was shown in the different scenarios proposed (Table 2). Additionally, classification made with the modified indices, show high correlation with dengue case occurrence, ranging between 70% and 85%, indicating its goodness as a predicting tool.

In this study, we could effectively classify the risk zones with only epidemiological data because we did not include entomological, climatic, socioeconomic variables, or environmental variables, which have been relevant in other studies to predict dengue occurrence [14,22,23,24,25,26]. The simplicity of this procedure allows implementation by health entities so that intervention decisions can be made quickly. Additionally, the data used are available for all places, and it is not necessary to procure other information that is difficult to access. 

Finally, the modified indices and methodology used in this research could be set to the finest temporal scales to build a permanent system of surveillance to determine, in real-time, zones for which it is mandatory to apply control strategies and monitor the effectiveness of these interventions. 

## 5. Conclusions

This study showed the necessity of modifying the risk indices proposed by [14] so that they could be used in areas during epidemic and endemic periods. The mathematical modification was achieved such that the risk measure scale is not affected by the dengue incidence of the area, due to the definition of epidemic being relative in the area. Therefore, the importance of the maximum value of the epidemiological wave for endemic scenarios was shown and indicated that a few successive weeks with dengue cases also represent a risk for a dengue epidemic.

If the dispersion of the disease from zone to zone is taken into account, LISA classification is more adequate for measuring risk than SD because it calculates risk based on the surrounding zones, whereas SD only allows for evaluating the risk of the zone for which it is being calculated.

Finally, we observed that using only epidemiological data, it is possible to determine the risk without introducing external variables such as climatic, entomological or environmental variables. The results of this risk determination could be used to generate early warning systems and to apply control measures to reduce the economic costs generated by dengue.

## Figures and Tables

**Table 1:** Risk measure using the LISA classification.

α_1_	β_1_	ϒ_1_ (×2)	Punctuation
L	L	L	4
H	L	L	5
L	H	L	5
L	L	H	6 *
H	H	L	6 *
H	L	H	7 *
L	H	H	7 *
H	H	H	8 *

Weights for the final LISA classification. For α_1_ and β_1_, we assigned 1 when the LISA classification indicated low risk (L) and 2 if LISA indicated high risk (H). Since ϒ_1_ comprises α_1_ and β_1_, we assigned 2 for L and 4 for H. The final risk classification was indicated by values equal to or greater than 6 (values marked with an asterisk).

**Table 2:** Hypothetical scenarios.

Hypothetical Scenarios	Zone	EW	EVmax	Risk Classification
A1	1	45	40	High
	2	40	40	Moderate
	3	45	40	High
	4	46	40	Very high
B1	1	50	25	Very high
	2	30	25	Moderate
	3	40	25	High
	4	50	25	Very high
	5	30	25	Moderate
	6	30	25	Low
	7	50	25	Very high
	8	30	25	Moderate
	9	30	25	Low
A2	1	30	48	High
	2	40	28	Very high
	3	50	25	High
	4	30	20	Moderate
B2	1	50	10	Moderate
	2	40	40	Very high
	3	40	40	High
	4	50	48	High
	5	30	28	Moderate
	6	30	25	Low
	7	50	25	Very high
	8	30	25	Moderate
	9	30	25	Low

Two vicinity configurations (A and B) were used to build hypothetical scenarios with the aim of validating the modified indices (Figure 3). We assumed a high incidence rate of dengue (100 cases per 100,000 people) and varied only the epidemiological weeks (EW) to obtain scenarios A1 and B1. Then, the maximum epidemiological wave (EVmax) was also varied to obtain scenarios A2 and B2. Risk classification in all scenarios demonstrated the ability of the modified indices to differentiate risk in areas with high dengue incidence.

**Table 3:** Correlation among dengue incidence and risk indices.

	α & α_1_	β	β_1_	ϒ	ϒ_1_
**2008**	0.92 **	0.08	0.90 **	0.20	0.32
**2009**	0.85 **	0.61 **	0.85 **	−0.06	0.81 **
**2010**	0.64 **	0.46 *	0.74 **	0.37 *	0.53 *
**2011**	0.88 **	0.46 *	0.93 **	−0.11	0.91 **
**2012**	0.97 **	0.91 *	0.99 **	0.18	0.88 **

Values of Pearson’s correlation coefficient between each index and dengue incidence in each area are shown. In general, correlation values for α_1_, β_1_, and γ_1_ are significantly high, whereas the older β and γ values exhibited low correlation values (* *p* < 0.05, ** *p* < 0.01).
